# Supplementary material for: Reconciling Mining with the Conservation of Cave Biodiversity: A Quantitative Baseline to Help Establish Conservation Priorities
Source: PLoS One. 2016 Dec 20;11(12):e0168348. doi: 10.1371/journal.pone.0168348 (PMC5173368; doi:10.1371/journal.pone.0168348)
Supplement: S1 Dataset — (ZIP) [file pone.0168348.s002.zip › Taxa/Serra Norte/SN_2013/PES/Lista PESL-01.pdf]

CAVIDADE PESL-0001

Classe	Ordem	Fam/Outros	Gên/Outros	Espécie	S-U	U-S	Zon.
Arachnida	Araneae	Pholcidae	*Mesabolivar*	*aurantiacus*	X	X	E
Arachnida	Araneae	Theridiosomatidae		sp.1	X		E
Arachnida	Opiliones	Sclerosomatidae	*Prionostema*	sp.1	X		E
Arachnida	Opiliones	Stygnidae	*Protimesius*	*gracilis*	12	20	E
Diplopoda	Polydesmida	Chelodesmidae		sp.1		1	E
Insecta	Diptera	Cecidomyiidae	Cecidomyiinae	sp.	X		E
Insecta	Diptera	Tipulidae	Tipulinae	sp.	X	X	E
Insecta	Heteroptera	Gerridae	*Brachymetra*	sp.		X	E
Insecta	Heteroptera	Veliidae	*Rhagovelia*	jovem	X		E
Insecta	Hymenoptera	Formicidae	*Camponotus*	sp.1		X	E
Insecta	Lepidoptera	Noctuoidea		sp.4		X	E
Turbellaria				sp.3		X	E
